# Are we looking under the lamp although we know the lost key is somewhere else? Or is it just about the egg?

**DOI:** 10.1080/03009734.2020.1755398

**Published:** 2020-05-18

**Authors:** Jan Holte, Thomas Brodin

**Affiliations:** a Carl von Linne Kliniken, Uppsala, Sweden;; b Department of Women’s and Children’s Health, Akademiska Sjukhuset, Uppsala University, Uppsala, Sweden

After more than 40 years with *in vitro* fertilization (IVF), our knowledge about which crucial factors determine human procreation, and how to best assist, is slowly increasing. Slowly, too slowly, is the slightly depressing conclusion Professor Georg Griesinger draws in the Editorial of this issue of *Upsala Journal of Medical Sciences*. Not even the flagship of unbiased evidence in medicine, randomized *controlled* trial (RCT), is left unaffected under his scrutiny. Most RCTs in assisted reproductive technologies (ART) are underpowered, and this problem is rarely solved by meta-analyses (1).

But how then do we improve our knowledge about which factors determine human procreation and hence improve diagnostic and treatment modalities? Needless to say, RCTs and relevant meta-analyses should be the main source of knowledge, but such studies always come after innovative and pilot research, with data suggesting improvements in the outcome of ART. Such pilot findings may be of the highest quality, like the invention of intracytoplasmic sperm injection (ICSI) (2), or the introduction of ultrasound guidance for oocyte collection (3), both methods almost immediately being introduced as game-changers. Importantly, in the case of ICSI, this method also provided the scientific society with the slightly surprising new information that male infertility in the majority of cases poses no problem when the oocytes are thus fertilized (4). This issue of *Upsala Journal of Medical Sciences* contains reviews of some new means and strategies for ovarian stimulation, i.e. random start (5), duo-stim (6), GnRH agonist trigger (7), and freeze-all (8) – all novelties with the potential of being true game-changers. Interestingly, like the new biologic information provided by ICSI, the random start of ovarian stimulation, used originally for urgent fertility preservation, also provides new knowledge about the endocrinology behind the recruitment of a dominant follicle in the human follicle phase (7). There is still a need for more carefully performed studies, however, for these novel methods and strategies to be established for appropriate patient populations. Questions remaining to be answered are: should all treatments be done with the freeze-all strategy, or only those at risk for OHSS-complications? Which protocol, if any, can safely be used for rescuing the luteal phase after a GnRH agonist trigger? Will a double trigger using both a GnRH agonist and hCG injections qualify as the new standard in the antagonist protocol in women with a low risk of OHSS?

The increasing use of adjuvant therapies in ART, most of which prove useless when seriously tested, is discussed in the paper by Nardo et al. (9). This is an important field, as the commercialization of ART promotes competition between clinics, a competition that often includes offers of various adjuncts. An analogy from the on-going coronavirus pandemic is the call from President Trump, on live TV, to use ‘hydroxychloroquine’ as a medication (4 April 2020) to treat the Covid-19 infection, much to the obvious embarrassment of his medical experts, as there is currently no evidence for benefits from such treatment.

In a brilliant lecture on evidence-based medicine at a recent conference in Sevilla, Professor Hans Evers, former Chief Editor of *Human Reproduction*, described eloquently the rise and the fall of one of these adjuvant therapies, the ‘endometrial scratch’. A few years ago, much to the surprise of many experienced clinicians with knowledge of the principles of human reproduction, it was suggested that this deliberate injury to the endometrium could improve implantation. The reports followed a well-known pattern: first ‘pilot’ papers with astonishingly positive results, based on retrospective case series (10). A few supporting papers followed, based on similar ‘preliminary’ data (11). The subsequent mental process in the clinical and scientific community was a feeling of positive surprise and hopefulness that a breakthrough was finally here for the notoriously depressing group of patients with recurrent implantation failure (RIF). In early and small publications, most often patients were their own controls, so that all included patients had one or more previous, failed treatments. After any intervention, here scratching, the post-intervention treatment will then – by pure chance – result in a pregnancy for some patients. This is a statistical phenomenon named ‘regression to the mean’, which describes the inevitable change to the better for the group as a whole, as no treatment could end worse than the first (12).

Based on the first positive results, many groups all over the world have introduced the method. The subsequent results are not uniformly positive, but it takes a while before a few negative reports are published (13–15) – it is more appealing to publish breaking news findings than dull negations of first reports, the well-known ‘publication bias’ (16). However, when later, often much later, the results from a correctly performed RCT are published (17), the bleak light of Monday morning strikes the enthusiastic clinicians advocating the method. The entire process from the first overestimated pilot results, through the intermediate sobering-up phase, to the disappointing RCT results follows an established curve called the Gartner hype curve or Gartner hype cycle. Sobering up is, however, not always the case. An interactive mentometer investigation during Hans Ever’s lecture revealed that two-thirds of the audience had offered, or still did offer, endometrial scratching.

As reviewed in the paper by Nardo et al. (9), there are numerous examples of similar developments of treatment innovations: addition of growth hormone, DHEA, corticosteroids, intralipid infusion, acupuncture, etc., all with more or less the same disappointing end results according to current evidence. Nevertheless, such negative results add significantly to our understanding of human procreation. Most of these methods are in the field of adjuvant therapies or adjuncts, i.e. based on a concept that factors outside the crucial gamete/embryo/uterus factor play a crucial role. Which ‘external’ factors remain to be examined? Immunological factors?

Until recently, it has been believed that the increased incidence of miscarriages, preeclampsia (18), and other complications related to placentation in donor oocyte recipient cycles (19) is caused by immunological incompatibility between the oocyte (embryo) and the recipient (20). New data, results from big data analyses, do, however, question this notion, as they suggest that a likely reason for such complications could be related to the lack of a corpus luteum, given that the endometrial preparation is commonly performed with hormone replacement therapy, also called programmed cycles, in oocyte donor–recipient cycles. Results from transfers of frozen–thawed embryos show that such pregnancy complications are increased in programmed cycles also in non-donor treatments, compared with transfers in ovulatory cycles (21). It seems likely that substances produced by the luteal body affect placentation in a way that a mere oestrogen/progesterone-supported endometrium usually does not achieve. These results thus shed new light on the basics of human fertility and ART. The results do not rule out the possibility of involvement of immunological factors *per se*, but they strongly suggest that factors produced by the corpus luteum, apart from progesterone, are important for a normal pregnancy.

The conclusions from the lack of positive effects on ART outcome from essentially all adjuvant therapies (9), combined with the excellent results of oocyte donation, are that the dominance of the ‘egg factor’ is becoming more evident for each year. The uterus and the endometrium definitely play ‘permissive roles’, i.e. pathological conditions such as intrauterine adhesions or submucous fibroids may reduce IVF results. Likewise, a notoriously thin endometrium lowers live birth rates (4). However, when such conditions are absent, does there remain a role for factors such as an unsynchronized endometrium in relation to embryo maturity? Although serious scientific and commercial efforts have been put into testing endometrial–embryo synchrony, such as the endometrial receptivity array (ERA)-test (22), there is little evidence today that this is a factor of true clinical importance. For severe uterine factors, surrogacy or uterine transplantation is the only option. In this issue of *Upsala Journal of Medical Sciences* the use of surrogacy is extensively discussed, focussing on the complicated ethical questions raised (23).

When one of the leading geneticists in preimplantation genetic testing for aneuploidies (PGT-a) was asked about the relative importance of the embryo versus the endometrium in human reproduction, his response was 90 to 10 in favour for the embryo, and a similar ratio for the oocyte versus the sperm, as a general estimate of the roles of the major players. Current results from PGT-A research support this notion (24). It is likely that with the progress of less invasive PGT-A-related technologies, increased accuracy of selection of the top embryo will take ART to higher success rates per transferred embryo. However, the legislation in some countries, like in Sweden, still does not allow PGT-A for selection of a euploid blastocyst in the absence of a known serious hereditary condition. The ethical problems associated with PGT-A as a means for embryo selection are discussed by Kjell Asplund (25). As reviewed by Lundin and Park, evidence-based assessments of the embryo cleavage and morphology in terms of scoring were introduced fairly late in the history of ART, and time-lapse monitoring has yet to prove its benefits (26). Will advanced analyses based on machine learning finally prove time-lapse technology to be worth the big costs (27)? Most likely, non-invasive PGT-A would provide an optimal means for embryo selection, and its use would reduce the treatment burden, helping with diagnostics in RIF cases and for patients with repeated miscarriages. Thus, this technology would be of value for a significant subgroup of patients in ART, and the ethical issues involved could most likely be dealt with by careful legislation.

The ‘egg factor’ is thus dominant as a cause of subfertility and the most frequently limiting factor. Recent evidence supports that ambitions to retrieve maximal numbers of oocytes per OPU should be encouraged, if needed with GnRH-agonist triggering (28). Although earlier data suggested that the pregnancy percentage decreases with oocyte numbers greater than 15–20 oocytes per retrieval (29), this is not true when additional FETs are included (28). Thus, ‘mild’ IVF stimulation has little role to play in modern IVF.

The ‘egg factor’ is still not well known among women in their 20 s. This is shown by studies that describe that young women tend to overestimate the fertility potential of women of 35–40 years of age, as reviewed by Delbaere et al. (30). For an increasing number of women, however, the possibility of storing vitrified oocytes for future use is seen as an option. So far, only few reports have been published on the outcome of warming and fertilizing such oocytes later. In this issue of *Upsala Journal of Medical Sciences* a review of pregnancy results in women returning for this purpose is given (31), showing indeed an age-factor for oocytes, with acceptable results for those who had their oocytes frozen before the age of 40, but not later.

Accumulating true new knowledge in the field of natural and assisted reproduction is a slow process and should be based on RCTs and big data analyses. Progress is hampered by numerous turns into blind alleys, each of which – if published – nevertheless contributes to current knowledge, as also negative results are important for the full picture. The oocyte is the crucial factor in most cases, which has to be kept in mind, when treatment and laboratory modalities are formed, and when patients are informed about the basic conditions estimating their chances for successful treatment.
